# An approach to and web-based tool for infectious disease outbreak intervention analysis

**DOI:** 10.1038/srep46076

**Published:** 2017-04-18

**Authors:** Ashlynn R. Daughton, Nicholas Generous, Reid Priedhorsky, Alina Deshpande

**Affiliations:** 1 Los Alamos National Laboratory, Los Alamos, 87544, USA

## Abstract

Infectious diseases are a leading cause of death globally. Decisions surrounding how to control an infectious disease outbreak currently rely on a subjective process involving surveillance and expert opinion. However, there are many situations where neither may be available. Modeling can fill gaps in the decision making process by using available data to provide quantitative estimates of outbreak trajectories. Effective reduction of the spread of infectious diseases can be achieved through collaboration between the modeling community and public health policy community. However, such collaboration is rare, resulting in a lack of models that meet the needs of the public health community. Here we show a Susceptible-Infectious-Recovered (SIR) model modified to include control measures that allows parameter ranges, rather than parameter point estimates, and includes a web user interface for broad adoption. We apply the model to three diseases, measles, norovirus and influenza, to show the feasibility of its use and describe a research agenda to further promote interactions between decision makers and the modeling community.

Despite substantial public health improvements in the last century, infectious diseases remain one of the leading causes of both morbidity and mortality^1^,^2^. When confronted with an infectious disease outbreak, public health officials typically work to control the outbreak by performing assessments, analyzing surveillance data, identifying resources and interacting with subject mater experts^2^,^3^,^4^. Control measures are then implemented based on the cumulative information collected. These approaches rely heavily on good surveillance systems, access to experts, and good intuition about which control measures to use. As such, they are largely subjective, time consuming, and the infrastructure required is often not present in high disease burden areas.

Modeling is an attractive supplemental method because of the ability to estimate an outbreak’s trajectory and the effects of possible control measures in a timely manner. Compartmental models are historically common; they divide individuals into categories based on their disease status. The most common variant is the SIR model, named after the categories used—“susceptible”, “infectious” and “recovered”. Models of this nature have small computational requirements, and are thus commonly used as first pass attempts to characterize outbreaks or infections quickly^5^. For example, after the sudden emergence of Severe Acute Respiratory Syndrome (SARS) in the early 2000s, researchers used modeling to characterize the virus’ epidemiology. Several used compartmental models with control measures like quarantine or isolation in various settings (e.g., hospitals or cities) to describe effects of possible interventions^6^,^7^,^8^,^9^. Similar work exists for essentially all well known infectious diseases. For example, Mandal *et al*. provide a review of models used for malaria^10^ and Bauch *et al*. explore model use with respect to SARS and other emerging infectious diseases^6^. Methods among these groups are often similar, but tend to focus on specific diseases and locations of interest.

In contrast, agent-based models use a bottom-up approach where the agents (these are often people) interact with particular rules to simulate outbreaks^11^. This allows simulations at high resolutions, but requires large amounts of data to parameterize the models, as well as substantial computational power. It is thought that they may reflect real world scenarios more accurately, but the lack of available epidemiological data necessitates assumptions that are difficult or impossible to test^11^. These models further require computational resources inaccessible to an average health department.

Both agent-based and compartmental models exhibit additional features that are problematic to wide-spread model adoption. The first is the focus on particular disease-location pairs. This emphasis precludes application to a new location or disease because of the amount of work associated with finding location specific data, tweaking parameters, and, often, reproducing code that is not freely available.

A related, but perhaps more pressing issue is the lack of collaboration between the researchers developing models and those making policy decisions during an outbreak. Wagner *et al*. describe the disconnect between these two fields^12^. The ultimate result is a lack of clarity in the modeling community about the requirements for real-world application of models and production of models that do not meet decision makers’ needs^12^,^13^.

There have been previous efforts to produce widely available models, in particular web-based simulations of agent-based disease models. Several of these efforts have been part of MIDAS (the Models of Infectious Disease Agent Study), including FRED^14^ and GLEAMviz^15^, which are both freely accessible and maintained. There is one package for the statistical language R^16^ that implements compartmental models. However, available web-based compartmental models are limited and are directed towards educating public health trainees rather than providing an operational modeling platform.

With this context in mind, our aim is to use existing models with low computational requirements to—(1) explore control measures and (2) develop an accessible platform for public health collaborators to use and provide feedback on models. For this initial work, we use a SIR model, modified to include a control measure, to explore many possible disease progression paths. The SIR model was chosen because it is the simplest and requires minimal computational resources. This study presents the application of existing SIR models to investigate control options using a “counterfactual”, a web application using the model, and a description of a path forward for validating SIR models.

A counterfactual is a theoretical construct describing a perfect experiment isolating one variable. For example, say patient *P* is in a clinical trial that is assessing the effects of drug *D* compared to a placebo. Patient *P*_*i*_ is randomly assigned to drug *D*. A perfect experiment would be for an identical patient, *P*_*j*_, to exist and be assigned to the placebo, so that the researchers could compare the differing effects of the drug versus the placebo in identical people. The concept of a counterfactual has existed in theory for decades^17^, and is commonly used in causal inference in medicine and epidemiology^18^. The counterfactual concept is not generally explicitly applied to epidemiological modeling, but some (e.g., Smith *et al*.^19^) mention the concept of exploring what “could” happen under different control scenarios. A policy maker could, for example, mentally compare an outbreak of strep throat where hand washing is the predominant intervention to an identical one where school closures are the main intervention and compare outbreak outcomes to aid in decision support about which intervention to use. This method aims to make those mental comparisons more explicit, thus affording more transparent decisions.

This work contributes to the field by providing a mechanism to weigh control measures (our *κ* value), an extensive description of ways to use the counterfactual in decision support, and a mechanism to enable broad adoption. By investigating plausible disease parameter ranges, rather than point estimates, we can analyze a number of possible outbreaks and begin to quantitatively understand how disease parameters affect outbreak outcomes of interest. Further, we contribute to the relative lack of compartmental model interfaces and provide a mechanism for iteration and feedback with public health end users.

To explore SIR models as decision support tools, we apply the model to three diseases—measles, norovirus and influenza. Within disease parameter ranges, we describe the worst case scenario where the model indicates a reduction in cumulative infected can still be achieved. These observations are hypothesis generating, rather than validated endpoints, because of the general lack of validation in previous work on SIR models. As such, we describe a path for future research in the discussion.

## Methods

### SIR model

As described above, the SIR model is a commonly used compartmental model used for infectious disease outbreaks. Because of the large literature base describing both the history and use of SIR models (e.g., see Keeling and Rohani^20^), we present a somewhat abbreviated description here.

**Figure 1:**
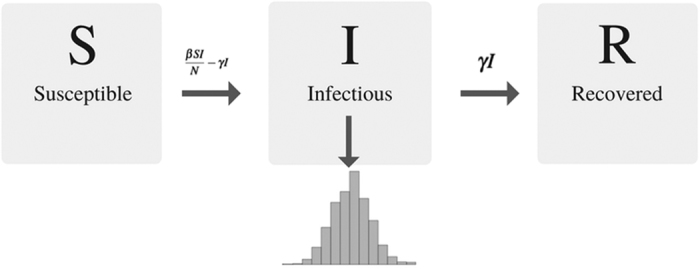
In a SIR model, individuals move between three compartments—S (susceptible), I (infectious) and R (recovered). Movement between the categories is dependent on *β* and *γ* which describe the “force” of the infection and the infectious period, respectively. The number of infectious persons at any given time results in the epidemic curve familiar to many epidemiologists.

People are assigned to three compartments based on their disease status at time *t* (see Fig. 1). The number of people in each compartment varies with time as the outbreak progresses, but the overall population in the model stays constant. Susceptibles (*S*) are those that are at risk of infection. Infected (*I*) are individuals experiencing the illness, and recovered persons (*R*) have completed infection and are now immune to the disease, or died as a result of the infection. Movement between compartments is described by the following system of equations:


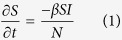



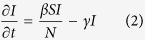



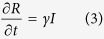



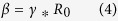



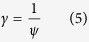


Transitions between compartments are described by two parameters, *γ* and *β. γ* is the reciprocal of *ψ*, the infectious period (see equation (5)). The infectious period is the interval of time during which an infected individual can transmit the disease. Time in our model is represented in days. Measles, for example, has an infectious period of approximately 8 days; individuals can transmit the disease from approximately 4 days before rash onset until 4 days after^21^. *γ* controls the transition from infectious to recovered (see equations (2) and (3)). *β* is commonly referred to as the “force of infection” because it describes how quickly a disease can move through a population. It is the product of *γ* and the reproductive number (*R*_0_), and controls the transition from susceptible to infectious (see equations (1) and (2)). The reproductive number, *R*_0_, is the number of secondary infections per primary infection (see equation (4)). Of note, *R*_0_ can be estimated a number of ways. Obadia *et al*.^22^ describe an overview of several common methods including exponential growth, maximum likelihood, sequential bayesian and a time-dependent method. Each method makes slightly different assumptions, and can result in different reproductive values. Values should be calculated and interpreted with these caveats in mind^23^.

### SIR augmentations

To expand the above model and introduce a scenario where a control measure is applied to an outbreak, we introduce two additional parameters:

1. *λ* or *control measure effectiveness* describes what fraction of individuals are removed from the susceptible population at each time point. For example, *λ* = 0 is a control that is completely ineffective and removes no individuals from the susceptible population during each time interval. Conversely, *λ* = 1 is a control measure that is 100% effective, or removes all susceptible individuals in one time interval. A more realistic value might be *λ* = 0.01, which would describe an intervention that removes 1% of the susceptible population during each time interval. A more detailed description of the appropriate interpretation of this parameter is included below.
2. *τ* or *control start* is the time unit (interpreted as days throughout this analysis) on which the control measure begins. For the purposes of the simulations presented here, it is assumed that the control is implemented on day *τ* and continued for the remainder of the outbreak.

To apply a control measure, equations (1) through (3) are modified such that if time (*t*) is ≥*τ*, a control measure with *λ* effectiveness is applied at each time step (see equations (6), (7), (8)). To denote the “controlled” environment the subscript *T* is added.


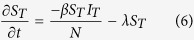



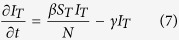



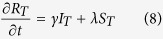


Within this implementation, *λ* is the fraction of the susceptible population removed at each time point. Operationally, this describes a control measure that eliminates the possibility of infection. For example, *λ* = 0.1 and *τ* = 5 indicates a scenario where 10% of the susceptible population is removed every day on and after the 5th day. This would describe an intervention like vaccination or quarantine. However, it does not describe the type of control measure that reduces infectivity, changes human behavior in a way that affects population density (e.g., staying home from work/school), or does not confer immunity to the infection (e.g., hand washing). Further, the control measure is continuously applied at each time point after initiation (e.g., 10% of the susceptible population are vaccinated each day for the remainder of the outbreak). This assumes that the control is implemented with the same effectiveness throughout each time interval. These assumptions simplify the addition of control measures to the SIR model, but it is relatively straightforward to modify this implementation in the future to a more realistic scenario. These equations are also limited to describing one control measure. It would be comparatively straightforward to substitute *λ* with a vector of parameters as opposed to a single term, in order to describe multiple control types and effectivenesses.

### Assumptions

There are a number of assumptions inherent in SIR models^24^. As a result, SIR models are scoped to diseases that meet the following criteria:

1. The disease is transmitted person-to-person. This means the disease is not transmitted via vector, or environmental component like water or food.
2. Disease transmission can be described via homogenous mixing. This means that if a group of susceptible people interact with an ill person, all susceptible person are equally likely to acquire infection. Of importance, a majority of infections do not meet this assumption. For example, sexually transmitted infections are not transmitted with equal probability among the entire susceptible population. Even in the case of airborne infections like measles this assumption ignores individuals’ specific immune responses (e.g., immunocompromised individuals are treated the same as healthy individuals).
3. The disease confers immunity. This means that once an individual has recovered they cannot get contract the illness again during the same outbreak. Diseases with very short-term (or no) immunity are commonly modeled with SI models.
4. The disease’s incubation period is relatively short. Diseases with long incubation periods should include the “exposed” category and can be modeled with a SEIR model^25^.
5. The disease is an acute illness (i.e., infected individuals recover or die). This excludes chronic diseases like hepatitis.

### *κ* - Comparing controlled and uncontrolled outbreaks

In order to compare a controlled outbreak to its counterfactual outbreak, we introduce the outcome measurement, *κ*. It describes the ratio of the cumulative number infected in a controlled outbreak to the cumulative infected in an uncontrolled outbreak. The cumulative number infected throughout the outbreak at time *t* is equal to the number recovered at time *t* plus the number infectious at that time (see equation (9)). At the end of an outbreak (*t* = *end*) there are no individuals left in the infected category and equation (9) reduces to equation (10).


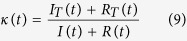



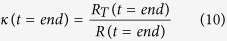


*κ* = 1 means that the controlled and uncontrolled outbreaks are identical, or that the control measure had no effect. *κ* > 1 means that the controlled outbreak had *more* infected than the uncontrolled outbreak (or that the control measure had a detrimental effect). *κ* < 1 indicates that the control measure reduced the number of infected persons. For example, *κ* = 0.01 is interpreted to mean that the controlled outbreak was 1% as large as the uncontrolled outbreak. *κ* = 0 would describe a scenario where the control measure stopped the outbreak from occurring at all.

### Sensitivity analysis

To assess the effect of each parameter on the model, we performed a sensitivity analysis. We varied *γ, β, λ* and *τ* within specific ranges (see Table 1) at random for 10,000 trials. During each trial, we randomly picked the value of each input parameter from the specified range, ran the model using those values, and recorded the outcomes. Here, outcomes of interest are the number infected in a controlled scenario, number infected in an uncontrolled scenario and the related *κ*. We then analyzed each parameter’s relative impact on these outcomes. Because *γ* and *β* vary together and can be described simultaneously using *R*_0_ (see equation (4)), we also analyzed how the change in *R*_0_ affects the outcomes. Because *λ* and *τ* only exist as parameters in controlled outbreaks, the controlled scenario is the only outcome considered.

### Application to three diseases

We applied this model to measles, norovirus, and influenza. These diseases were selected because they are of public health interest, and because they meet the requirements described in the assumptions section above. Of note, norovirus can be transmitted both through food and via person-to-person. Outbreaks described here are solely *person-to-person* transmitted outbreaks.

Outbreaks were simulated using standard parameter ranges for the three diseases. These ranges were identified based on literature values reported for parameters, identified via searches using Google Scholar and PubMed. Search terms included “[disease name] + infectious period”, “[disease name] + contact rate”, “[disease name] + force of infection”, and “[disease name] + reproductive number”. We were consistently unable to find reported literature values for *β* and instead used equation (5) to find the maximum and minimum *β* values for each disease, given their infectious period and *R*_0_ values.

Rather than attempting to identify control parameters (*λ* and *τ*) based on literature values, we intentionally selected a broad range of possible parameters to observe the effects of a broad number of controls. To account for the logistic work that precedes control initiation (identifying the outbreak, laboratory confirmation, mobilizing resources etc.) we selected a minimum control start of 3 days. We then selected upper bounds based on typical outbreak progression for each disease. Measles and influenza can both result in outbreaks that are several weeks to months long. Thus, we selected one month (30 days) as the upper bound of *τ*. Conversely, norovirus outbreaks are typically much shorter due to their short infectious period. We thus limited the latest possible control start to 7 days. All *λ* values were varied between 0.005 and 0.3 (0.5% to 30%). Table 2 describes the ranges used for each parameter and disease.

### Development of a web-based tool

To make this model available for decision making, we developed a web-based application that allows a user to enter parameter ranges for their disease, initial population variables and control information. It is a Django application^26^ that uses HighCharts^27^ for visualization. All code for the SIR model was written in Python 3.5^28^.

To make the application more user friendly, two small modifications were made to *γ* and *β* such that they could be expressed as the infectious period (see equation (11)) and *R*_0_ (see equation (12)). These terms are more familiar to public health individuals than *γ* and *β*, which are commonly used by modelers.


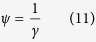



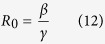


## Results

### Sensitivity analysis

**Figure 2:**
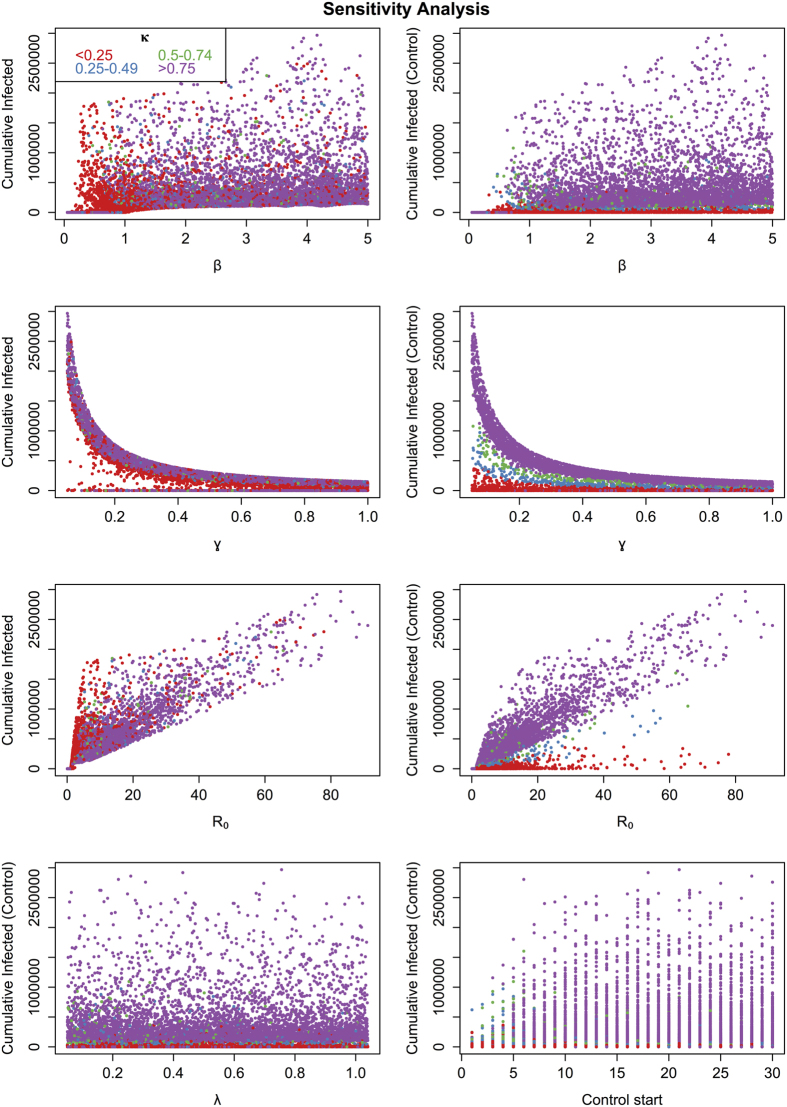
Each scatterplot shows a parameter (*β, γ, λ, R*_0_ or *τ*) with respect to outbreak size, represented here by the cumulative number of infected individuals in a controlled or uncontrolled outbreak. Each point in the scatterplot corresponds to one trial, as described in the text. Points are colored by the range in which the corresponding *κ* score falls. Here, strong relationships between parameters and the outcome (e.g., *γ* and *R*_0_) indicate stronger influence on outbreak size compared with parameters that have weak or no relationships (e.g., *β, τ*).

Figure 2 shows the results of the sensitivity analysis. Plots show each parameter with respect to the cumulative number infected (in either controlled or uncontrolled outbreaks), and are colored by the range of the associated *κ* score. *γ* shows a strong negative correlation with the cumulative number infected (i.e., larger *γ* values (shorter infectious periods) result in smaller outbreaks) and *R*_0_ shows a positive association with the number of persons affected (i.e., more quickly moving outbreaks infect more people). Both correspond to our intuition about outbreak progression—diseases with short infectious periods infect fewer individuals because the disease is infectious for less time and can thus spread to fewer persons. Conversely, large *R*_0_ values correspond to situations where the host can infect many other people, thus resulting in much larger outbreaks.

Results further indicate that *β, λ* and *τ* affect overall outbreak size substantially less. There is a weak association between *β* and outbreak size in controlled outbreaks, as well as a possible association between *β* and *κ*, but essentially no association between *λ* and outbreak size or *λ* and *κ*.

### Disease application

**Figure 3:**
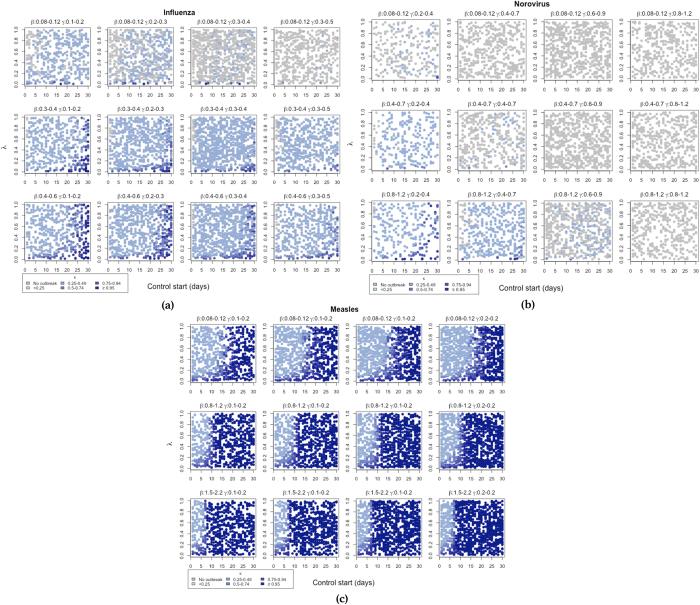
Three sets of scatterplots describing simulated outbreaks of influenza, norovirus and measles are shown. Individual scatterplots provide a cross section of possible outbreaks where rows hold *β* ranges constant while columns hold *γ* ranges constant. Plots show *λ* values on the y-axis, control start (*τ*) values on the x-axis and are colored by *κ* ranges. Each point denotes a counterfactual (i.e., a controlled outbreak within the given *β* and *γ* ranges compared to an identical uncontrolled outbreak). The color indicated the *κ* score associated with the counterfactual trial. Gray points are combinations that yield no outbreak (defined here as fewer than 2 cases overall), and progressively darker shades of blue indicate less difference between the controlled and uncontrolled outbreak.

Figure 3 describes a number of outcomes for measles, norovirus and influenza outbreaks based on literature parameter values (see Table 2) and the resulting *κ*. Patterns are recognizable both within and across diseases. Within norovirus, for example, it is evident that there are several combinations of outbreaks that produce no outbreak (here defined as fewer than 2 cases total—see gray dots). In particular, as *γ* approaches larger values (>0.3) individuals progress from infected to recovered too quickly to pass the illness to others. This is consistent with many point source norovirus outbreaks where the number of secondary cases is generally quite small.

Conversely, the vast majority of measles outbreaks simulated are essentially unaffected by any control measure tested (see dark blue dots that indicate controlled and uncontrolled outbreaks are ≥95% similar). Within a given cross-section of outbreak parameters, the *τ* value (control measure start) affects the resulting *κ* more than *λ* indicating that, under this model, implementing a control measure early is more important than implementing the most effective control measure. This is a potentially important finding for decision support and is an intriguing path for further investigation. It is also consistent with our sensitivity analysis findings (see Fig. 2).

For each disease, we identify the latest possible control start and the least effective intervention that could still result in *κ* values of 0.1 and 0.01 (see Table 3). Interestingly, if control measures have *λ*s that are large enough (minimum 5%), or control starts that are early enough (6—30 days) they can consistently produce dramatic reductions in outbreak load. By examining these values in various parameter ranges, we can begin to see the effects of parameter ranges on *κ* results. In Table 3, we consider (1) the entire range, (2) the lower 50th percentile of both *γ* and *β* values or (3) the upper 50th percentile of both *γ* and *β* values. There is a strong distinction between outbreaks with large values (upper 50th percentile) compared to outbreaks with small values. For example, in measles outbreaks, although it is possible to reduce the outbreak to 10% of the uncontrolled outbreak by beginning a control measure 29 days after outbreak onset, dividing the outbreaks into upper and lower 50th percentiles indicates this is actually only possible if both the *β* and *γ* parameters fall into the lower 50th percentile and the control is at least 19% effective. Similar, but less dramatic trends are evident in norovirus and influenza.

These examples illustrate the possible use of models like this for decision support. By aggregating several models, it is possible to identify general trends that are relevant for intervention decisions.

### User interface

**Figure 4:**
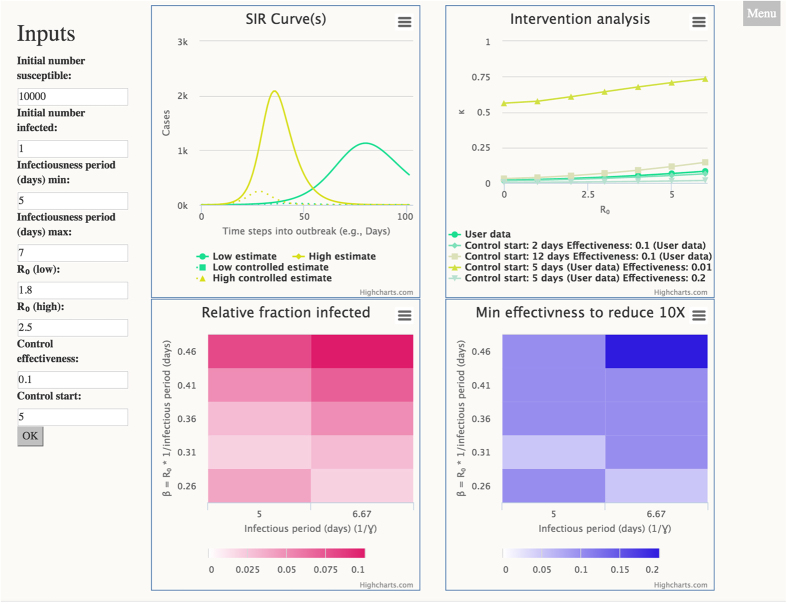
A web-based application using the model presented is shown. User input fields are on the left, and application output is on the right. Inputs are simple, and allow the user to describe ranges of parameters rather than point estimates. Application outputs include traditional epidemiology curves (top left), simple line charts describing the effect of changing control starts or effectiveness (top right) and two heat maps to describe the impact of various parameters on the resulting outbreak (bottom row).

To facilitate widespread use of the model, a user interface was developed. Figure 4 shows an example of user data and application output. Output includes the smallest and largest SIR curves possible based on user input, as well as the effect of user control measures on those curves. An additional three graphs describe how outputs (*κ*) change with changing *R*_0_, *β* and *γ* values, and describe the minimum required control effectiveness to reduce the outbreak ten times. Visualizing the data multiple ways allows the user to see different aspects of the same outbreak, and facilitates enhanced decision making capabilities. In the example presented, the second graph (titled ‘Intervention analysis’) indicates that changing the control start date by a few days in either direction minimally impacts the resulting *κ* score, regardless of the *R*_0_ value. However, changing control effectiveness from 0.01 to 0.1 dramatically increases *κ*.

## Discussion

We conducted this study to evaluate the feasibility of a simplified approach to decision support for control measure intervention. The larger goal is the development of methodologies that improve collaboration between public health and modeling communities which in turn can facilitate optimum disease response during outbreaks.

Our results suggest that it is reasonable to simultaneously explore the impact of a variety of control measures on outbreak progression in a number of scenarios using simple SIR models. We do so while using a range of outbreak parameters, to understand the effects of both outbreak parameters and control efforts on outbreak progression. We show that, in this model, *γ* affects the outbreak outcomes most substantially. We further provide a way to measure the relative success of outbreak control using the *κ* value. We lastly present one possible method to promote adoption of models in the public health community by presenting a simple, web-based interface for the model.

Compartmental models are in many ways preferable to agent-based models because of their simplicity and small computational requirements. However the use of SIR models necessitates adoption of several assumptions that rarely exist in real world outbreaks. Of particular concern is the assumption of homogenous mixing. However, there are numerous ways to improve upon the simple model described here. Other compartmental models (e.g., SEIR, SIS, SI etc.) and methods exist to reduce or modify these assumptions and expand the breadth of applicable disease. For example, Hethcote *et al*. describe a method to allow non-homogenous mixing within compartmental models for sexually transmitted infections^29^.

Another possibility is the addition of an underlying network to improve model behavior. For example, Meyers *et al*.^30^ found that coupling a compartmental model with an underlying social network allowed them to explain aspects of real SARS epidemics (used for illustration purposes in the introduction) better, than the compartmental models alone. Related possibilities include additions of spatial networks in addition to or instead of social networks^31^. It is possible that various networks are suited to particular diseases or disease scenarios. These subtleties offer opportunities for extensive further research. Importantly, many variations of these models continue to maintain comparatively low computational requirements, while allowing for a better representation of reality.

Another, related focus should be continued research on the impact of parameter selection on model outputs. Here, we describe an approach where parameters are assumed to be known (or estimate-able), and the range of possible outbreaks are treated as an outcome. In contrast, Wearing *et al*. estimate parameters by finding the best simulated outbreak fit to real data and identifying the parameters that give rise to that simulation^32^. Their results caution that model selection (e.g., the type of compartmental model used) can dramatically affect the resulting reproductive ratio estimated. Our results indicate that, in addition, variations in reproductive ratio produce exceedingly different outbreaks. Meyers *et al*.^30^ also note the large impact parameter selection and network structure can have on resulting simulated outbreaks.

One obvious possible improvement is in the continued production and extension/refinement of tools to utilize compartmental models and afford control measure simulation quickly and easily. The tool presented here, for example, might be enhanced by adding new compartmental models, refining control definitions, improving visualization, and investigating addition of network structures. Deployment of these systems as open-source code, or freely available web applications should be encouraged.

Overall, there is a clear need in the field to better understand outbreak parameters, model selection, underlying model assumptions, and the ways that these apply to real world scenarios. While SIR models have been used extensively for many years, there has been little work done on validating their output. We thus propose thoughtful validation of SIR models as an important next step. One method to accomplish this is to compare the outputs of validated agent-based models to outbreaks produced using compartmental models. Previously validated agent-based models simulating disease outbreak progression on a fine tuned scale already exist (e.g., EpiSimS^33^,^34^) and would provide good candidates for this research.

Such a validation would accomplish several things. It would (1) validate the counterfactual approach, (2) provide additional data to describe when compartmental models are appropriate approximations of real world outbreaks and (3) provide data to describe situations where the compartmental models do not match real world outbreaks and should not be used for decision support.

## Additional Information

**How to cite this article:** Daughton, A. R. *et al*. An approach to and web-based tool for infectious disease outbreak intervention analysis. *Sci. Rep.* **7**, 46076; doi: 10.1038/srep46076 (2017).

**Publisher's note:** Springer Nature remains neutral with regard to jurisdictional claims in published maps and institutional affiliations.

## Figures and Tables

**Table 1:** Sensitivity analysis ranges tested.

Parameter	Range tested (units)	Controlled or uncontrolled outcome
*γ*	0.05–1.0 (days^−1^)	Both
*β*	0.05–5.0 (unitless)	Both
*R*_0_	1.0–100 (unitless)	Both
*λ*	0.01–1.0 (fraction of susceptibles removed)	Controlled only
*τ*	1–31 (days)	Controlled only

**Table 2:** Parameter Selection.

Disease	Infectious period (days)	*γ*	*R*_0_ values	*β* values	*λ*	*τ* (days)
Measles	8^21^	0.1–0.2	1–18^35^,^36^,^37^,^38^	0.1–3.6	0.5–30%	3–30
Norovirus	0.5–14^39^	0.07–2	1.6–7.3^40^,^41^	0.1–2.0	0.5–30%	3–7
Influenza	3–7^42^	0.1–0.4	0.9–2.27^43^,^44^	0.1–0.9	0.5–30%	3–30

**Table 3:** Control measure summary results.

	*β* and *γ* range percentile	Measles	Norovirus	Influenza
Latest *τ* for *κ* ≤ 0.1 (associated *λ*)	All	29 (19%)	6 (5%)	29 (15%)
Latest *τ* for *κ* ≤ 0.1 (associated *λ*)	Lower 50%	29 (19%)	6 (5%)	29 (17%)
Latest *τ* for *κ* ≤ 0.1 (associated *λ*)	Upper 50%	None	6 (16%)	29 (29%)
Smallest *λ* for κ ≤ 0.1 (associated *τ*)	All	5% (6)	5% (6)	5% (12)
Smallest *λ* for *κ* ≤ 0.1 (associated *τ*)	Lower 50%	7% (24)	5% (6)	5% (6)
Smallest *λ* for *κ* ≤ 0.1 (associated *τ*)	Upper 50%	None	6% (5)	6% (10)
Latest *τ* for *κ* ≤ 0.01 (associated *λ*)	All	29 (27%)	6 (5%)	29 (7%)
Latest *τ* for *κ* ≤ 0.01 (associated *λ*)	Lower 50%	29 (19%)	6 (9%)	29 (17%)
Latest *τ* for *κ* ≤ 0.01 (associated *λ*)	Upper 50%	None	6 (16%)	19 (29%)
Smallest *λ* for *κ* ≤ 0.01 (associated *τ*)	All	7% (24)	5% (6)	5% (12)
Smallest *λ* for *κ* ≤ 0.01 (associated *τ*)	Lower 50%	7% (24)	6% (5)	6% (9)
Smallest *λ* for *κ* ≤ 0.01 (associated *τ*)	Upper 50%	None	6% (5)	13% (5)

## References

[b1] Lopez A. D. . Global and regional burden of disease and risk factors, 2001: systematic analysis of population health data. The Lancet 367, 1747–1757, doi: 10.1016/S0140-6736(06)68770-9 (2006). 16731270

[b2] Abdallah S. & Panjabi R. Control of communicable diseases. In Public health guide in emergencies 284–369, 2 edn. http://www.jhsph.edu/research/centers-and-institutes/center-for-refugee-and-disaster-response/publications_tools/publications/_CRDR_ICRC_Public_Health_Guide_Book/Pages_from_Chapter_7_.pdf. (Johns Hopkins Bloomberg School of Public Health, 2008).

[b3] Murray C. K. . An Approach to Prevention of Infectious Diseases during Military Deployments. Clinical Infectious Diseases 44, 424–430, doi: 10.1086/510680 (2007). 17205453

[b4] Frieden T. R. . A CDC framework for preventing infectious diseases - Sustaining the essentials and innovating for the future. Tech. Rep. Centers for Disease Control and Prevention (CDC) (2011) http://www.cdc.gov/oid/docs/id-framework.pdf (Date of access: 7/27/16).

[b5] Brauer F. . (eds) Mathematical Epidemiology, vol. 1945 of Lecture Notes in Mathematics (Springer, Berlin Heidelberg, 2008). http://link.springer.com/10.1007/978-3-540-78911-6.

[b6] Bauch C. T. . Dynamically Modeling SARS and Other Newly Emerging Respiratory Illnesses: Past, Present, and Future. Epidemiology 16, 791–801, doi: 10.1097/01.ede.0000181633.80269.4c (2005). 16222170

[b7] Lloyd-Smith J. O., Galvani A. P. & Getz W. M. Curtailing transmission of severe acute respiratory syndrome within a community and its hospital. Proceedings of the Royal Society B: Biological Sciences 270, 1979–1989, doi: 10.1098/rspb.2003.2481 (2003). 14561285 PMC1691475

[b8] Gumel A. B. . Modelling strategies for controlling SARS outbreaks. Proceedings of the Royal Society B: Biological Sciences 271, 2223–2232, doi: 10.1098/rspb.2004.2800 (2004). 15539347 PMC1691853

[b9] Nishiura H. . Modelling potential responses to severe acute respiratory syndrome in Japan: the role of initial attack size, precaution, and quarantine. Journal of Epidemiology and Community Health 58, 186–191, doi: 10.1136/jech.2003.014894 (2004). 14966229 PMC1732706

[b10] Mandal S., Sarkar R. & Sinha S. Mathematical models of malaria - a review. Malaria Journal 10, 202, doi: 10.1186/1475-2875-10-202 (2011). 21777413 PMC3162588

[b11] An L. Modeling human decisions in coupled human and natural systems: Review of agent-based models. Ecological Modelling 229, 25–36, doi: 10.1016/j.ecolmodel.2011.07.010 (2012).

[b12] Wagner W. E., Fisher E. C. & Pascual P. Misunderstanding Models in Environmental and Public Health Regulation. SSRN Scholarly Paper ID 1711766, Social Science Research Network (2010) http://papers.ssrn.com/abstract=1711766.

[b13] Margevicius K. J. . The Biosurveillance Analytics Resource Directory (BARD): Facilitating the Use of Epidemiological Models for Infectious Disease Surveillance. PLOS ONE 11, e0146600, doi: 10.1371/journal.pone.0146600 (2016). 26820405 PMC4731202

[b14] Grefenstette J. J. . FRED (a Framework for Reconstructing Epidemic Dynamics): an open-source software system for modeling infectious diseases and control strategies using census-based populations. BMC public health 13, 940, doi: 10.1186/1471-2458-13-940 (2013). 24103508 PMC3852955

[b15] den Broeck W. V. . The GLEaMviz computational tool, a publicly available software to explore realistic epidemic spreading scenarios at the global scale. BMC Infectious Diseases 11, 37, doi: 10.1186/1471-2334-11-37 (2011). 21288355 PMC3048541

[b16] Jenness S., Goodreau S. & Morris M. EpiModel (2015) https://doi.org/10.5281/zenodo.16767 (Date of access: 11/30/16).

[b17] Lewis D. Counterfactual dependence and time’s arrow. Noûs 13, 455–476, doi: 10.2307/2215339 (1979).

[b18] Höfler M. Causal inference based on counterfactuals. BMC Medical Research Methodology 5, doi: 10.1186/1471-2288-5-28 (2005). PMC1239917 16159397

[b19] Smith T. . Mathematical Modeling of the Impact of Malaria Vaccines on the Clinical Epidemiology and Natural History of Plasmodium Falciparum Malaria: Overview. The American Journal of Tropical Medicine and Hygiene 75, 1–10 (2006). http://www.ajtmh.org/content/75/2_suppl/1. 10.4269/ajtmh.2006.75.2_suppl.0750001 16931810

[b20] Keeling M. J. & Rohani P. Modeling Infectious Diseases in Humans and Animals (Princeton University Press, 2008).

[b21] Measles investigation quicksheet (2016) https://www.cdph.ca.gov/programs/immunize/Documents/CDPHMeaslesInvestigationQuicksheet.pdf (Date of access: 7/12/2016).

[b22] Obadia T., Haneef R. & Boëlle P.-Y. The R0 package: a toolbox to estimate reproduction numbers for epidemic outbreaks. BMC Medical Informatics and Decision Making 12, 147, doi: 10.1186/1472-6947-12-147 (2012). 23249562 PMC3582628

[b23] Breban R., Vardavas R. & Blower S. Theory versus Data: How to Calculate R0? PLoS ONE 2, e282, doi: 10.1371/journal.pone.0000282 (2007). 17356693 PMC1804098

[b24] Beretta E. & Takeuchi Y. Global stability of an SIR epidemic model with time delays. Journal of Mathematical Biology 33, doi: 10.1007/BF00169563 (1995). 7897328

[b25] Li M. Y. . Global dynamics of a SEIR model with varying total population size. Mathematical Biosciences 160, 191–213, doi: 10.1016/S0025-5564(99)00030-9 (1999). 10472754

[b26] Django (2013) https://djangoproject.com (Date of access: 8/9/16).

[b27] HighCharts (2016) http://www.highcharts.com/ (Date of access: 8/23/2016).

[b28] Python (2016) https://www.python.org/ (Date of access: 8/17/16).

[b29] Hethcote H.W., Yorke J. A. & Nold A. Gonorrhea modeling: a comparison of control methods. Mathematical Biosciences 58, 93–109, doi: 10.1016/0025-5564(82)90053-0 (1982).

[b30] Meyers L. A. . Network theory and SARS: predicting outbreak diversity. Journal of Theoretical Biology 232, 71–81, doi: 10.1016/j.jtbi.2004.07.026 (2005). 15498594 PMC7094100

[b31] Sun G.-Q. . Pattern transitions in spatial epidemics: Mechanisms and emergent properties. Physics of Life Reviews, doi: 10.1016/j.plrev.2016.08.002 (2016). PMC7105263 27567502

[b32] Wearing H. J., Rohani P. & Keeling M. J. Appropriate Models for the Management of Infectious Diseases. PLoS Medicine 2, e174, doi: 10.1371/journal.pmed.0020174 (2005). 16013892 PMC1181873

[b33] Fairchild G. . Optimizing human activity patterns using global sensitivity analysis. Computational and Mathematical Organization Theory 20, 394–416, doi: 10.1007/s10588-013-9171-0 (2014). 25580080 PMC4286349

[b34] Mniszewski S. M. . Pandemic simulation of antivirals + school closures: buying time until strain-specific vaccine is available. Computational and Mathematical Organization Theory 14, 209–221, doi: 10.1007/s10588-008-9027-1 (2008). PMC7087848 32214872

[b35] Keeling M. J. Disease Extinction and Community Size: Modeling the Persistence of Measles. Science 275, 65–67, doi: 10.1126/science.275.5296.65 (1997). 8974392

[b36] Bjornstad O. N., Finkenstadt B. F. & Grenfell B. T. Dynamics of Measles Epidemics: Estimating Scaling of Transmission Rates Using a Time Series SIR Model. Ecological Monographs 72, 169, doi: 10.2307/3100023 (2002).

[b37] Bonačić Marinović A. A. . Effectiveness and Timing of Vaccination during School Measles Outbreak. Emerging Infectious Diseases 18, 1405–1413, doi: 10.3201/eid1809.111578 (2012). 22931850 PMC3437694

[b38] WHO guidelines for epidemic preparedness and response to measles outbreaks. Tech. Rep. WHO/CDS/CSR/ISR/99.1, Department of Communicable Disease Surveillance and Response (CSR), World Health Organization (1999) http://www.who.int/csr/resources/publications/measles/whocdscsrisr991.pdf. (Date of access:7/28/16).

[b39] Norovirus (2016) http://www.cdc.gov/norovirus/about/transmission.html (Date of access: 7/12/2016).

[b40] Dye C. Epidemiology: Modeling the SARS Epidemic. Science 300, 1884–1885, doi: 10.1126/science.1086925 (2003). 12766208

[b41] Simmons K. . Duration of Immunity to Norovirus Gastroenteritis. Emerging Infectious Diseases 19, 1260–1267, doi: 10.3201/eid1908.130472 (2013). 23876612 PMC3739512

[b42] Influenza Infection: CDNA National Guidelines for Public Health Units (2011). http://www.health.gov.au/internet/main/publishing.nsf/Content/3D622AEAE44DDEB2CA257BF0001ED884/$File/Influenza-SoNG-july11.pdf (Date of access: 7/12/2016).

[b43] Coburn B. J., Wagner B. G. & Blower S. Modeling influenza epidemics and pandemics: insights into the future of swine flu (H1N1). BMC Medicine 7, 30, doi: 10.1186/1741-7015-7-30 (2009). 19545404 PMC2715422

[b44] Biggerstaff M. . Estimates of the reproduction number for seasonal, pandemic, and zoonotic influenza: a systematic review of the literature. BMC Infectious Diseases 14, doi: 10.1186/1471-2334-14-480 (2014). PMC4169819 25186370

