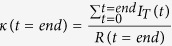# Corrigendum: An approach to and web-based tool for infectious disease outbreak intervention analysis

**DOI:** 10.1038/srep46852

**Published:** 2017-06-19

**Authors:** Ashlynn R. Daughton, Nicholas Generous, Reid Priedhorsky, Alina Deshpande

Scientific Reports 7: Article number: 4607610.1038/srep46076; published online: 04 18 2017; updated: 06 19 2017

This Article contains errors in the following equations:

In Equation 9,


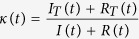


should read:


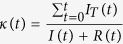


In Equation 10,


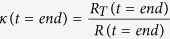


should read: